# Foreign Summary

**Published:** 1840-02-08

**Authors:** 


### FOREIGN SUMMARY.

### Velpeau's Clinical Lecture on Ophthalmia.—No. xvi.

*Non-existence of scrofulous ophthalmia as a specific disease.—Truly specific nature of syphilitic ophthalmia.*

*Scrofulous Ophthalmia.*

A modern writer thus commences the chapter in which he treats of scrofulous ophthalmia:—"If there are persons to be found sufficiently sceptical to doubt that an inflammatory affection of the eye may undergo peculiar modifications attributable to the causes which have produced that affection, the arguments which scrofulous ophthalmia furnishes will surely convince them of their error." To this I would answer, as I have repeatedly done, that no one doubts the influence which certain causes exercise over disease, nor do I mean in any way to deny it. My views may be expressed in a very few words; I do not believe that the symptoms of inflammatory diseases of the eyes are essentially modified in scrofulous subjects, or that the indications with regard to treatment are essentially different.

The arguments which the followers of the German school bring forward to support their opinions, are drawn, firstly, from the peculiar symptoms which they suppose scrofulous ophthalmia to present; and, secondly, from the treatment which it requires. M. Sichel recognises two forms of scrofulous ophthalmia: *scrofulous keratitis.* To these he also adds *scrofulous blepharitis.*

*Scrofulous conjunctivitis* is characterized by partial vascularization of that portion of the conjunctiva which is near the commissure of the eyelids. This vascularization is formed by streaks of a pale red colour, and represents a triangle, the base of which is turned towards the cornea, and the summit towards the internal or external canthus. Sometimes at the base of this triangle, papulæ or pustules of variable size are observed. The sclerotica remains perfectly white underneath. There is neither photophobia nor epiphora.

Such are the symptoms which are usually attributed to scrofulous conjunctivitis. If, however, you will analyse them, you will perceive that they are exactly the same as those which I described to you in a former lecture as belonging to papular or partial conjunctivitis,—a disease which is met with as frequently on persons who are not scrofulous as on those who are. If any here still retain doubts on the subject, let them examine several patients now in our wards, and they will at once become convinced of the truth of what I now assert.

In *scrofulous keratitis*, although the cornea never becomes vascularized, it loses its transparency, and becomes cloudy. Effusion of plastic lymph may also take place in the tissue of the membrane, the surface of which then appears uneven, as if covered with sand, and assumes a grayish colour.

On consideration you will find that these symptoms, the most prominent among those which are attributed by authors to "scrofulous keratitis," belong in reality to the various forms of simple inflammation of the cornea, which I described to you a short time ago. You will, indeed, observe them quite as often in persons who do not present the slightest trace of a scrofulous constitution, as in those who are scrofulous—a fact, the correctness of which I have frequently proved at the bedside of the patient.

The same remarks will also apply to *scrofulous blepharitis*, which is nothing more than the affection of the eyelids which I described to you under the name of glandular blepharitis.

But although I consider the symptoms which are generally supposed to characterize these specific affections as merely indicating simple inflammation of one or more of the membranes of the eye, I at the same time believe that the progress and duration of an inflammatory affection of the eyes is always more or less modified when it attacks a person whose constitution is deeply tainted with scrofula. In these instances the general state of the economy seems to impress on the ocular inflammation a kind of chronicity, so that effectually to subdue the disease and to prevent the relapses which are continually occurring, it is absolutely necessary to act on the constitution. But this alone does not certainly authorize our making specific affections of the ophthalmia which occurs on scrofulous subjects. Does not, indeed, a general scrofulous taint exercise the same influence over the course and duration of all other diseases? How is it, then, that scrofulous ophthalmia has been so universally recognised, not only by ophthalmologists, but also by the profession in general? This circumstance at first appears extraordinary, but it may easily be accounted for. Let us suppose that a practitioner meets with an ophthalmia, presenting the symptoms which I have enumerated in a scrofulous subject, and that shortly after he meets with several other scrofulous individuals, labouring under the same form of inflammation. Unless he examines the question as we are now examining it, he will probably feel perfectly warranted in establishing a scrofulous form of inflammation. When he has once come to the conclusion that such a disease really exists, his belief will not be staggered by meeting with cases which present the same symptoms, although there is nothing visibly scrofulous in the constitution of the patient. On the contrary, he will say that as his patient is affected with scrofulous ophthalmia, and no symptom of scrofula can be discovered, the scrofula must be in a latent state. A fact which proves at once the absurdity of such reasoning is, that this form of ophthalmia attacks persons of a sanguineous as often as those of a lymphatic constitution. I have, indeed, met with it in persons of every age, and of every temperament. Scrofulous individuals themselves are liable to be attacked by all the various forms of inflammation which I have described, the symptoms varying according to the nature of the tissue which is inflamed.

In my opinion, even when no allusion is made to diseases of the eye, the word "scrofulous" is very much abused, many persons being reputed scrofulous, although, in reality, they are perfectly free from such an affection. Indeed, in the present state of science, whenever a person has tumefied indurated glands in the neck, or underneath the lower maxilla, he is called "scrofulous," although his flesh may be firm, his shoulders wide, his complexion highly coloured, and he has only become so from having received a scratch or some other trifling injury. It is really preposterous to call a strong robust person scrofulous because he happens to have had an inflammatory affection of the lymphatic system. Properly speaking, there is no such constitution as a scrofulous constitution. When this expression is used, we merely suppose that by it is meant a lymphatic constitution, in which the white fluids, predominating over the others, predispose to diseases of the lymphatic system.

In speaking of "rheumatic ophthalmia," I told you that the belief in the specific nature of the disease was prejudicial in a therapeutic point of view; this may also be said with regard to scrofulous ophthalmia. Thus, you will find that those who follow the ideas which I now oppose, consider general measures, and more especially those which are commonly employed in the treatment of scrofula, as principally indicated, not only with really scrofulous subjects, but also whenever the eye presents the characters which are supposed to belong to this form of inflammation. In my wards I have long followed, with great success, a very different plan of treatment. I treat the inflammation locally by the application of astringents, and have seldom recourse to general measures, unless the general state of the patient seems to demand it. When there is any constitutional predisposition, such as scrofula—for I consider scrofula to be nothing more than a constitutional predisposition—I always have recourse to those therapeutic agents which are calculated to modify it.

In fine, scrofulous ophthalmia may be considered a nonentity that ought to be entirely rejected from nosological classifications, which it merely renders more complex without any practical utility.

From the remarks which I have made in this and in the preceding lecture, it is evident that ophthalmology would be much simplified were all these specific forms of ophthalmia definitively abandoned, and the inflammatory diseases of the eyes merely studied in the tissues which are affected. That you may become convinced of the great advantage that would ensue, both to science and to therapeutics, I need only to ask you to look into the works of the authors who have written on this branch of pathology; you will really be alarmed at the confusion—the chaos which reigns in them. In these lectures I have merely laid before you the doctrine of specific ophthalmia in its greatest simplicity, and yet you must have remarked that the lucidity which characterized the description which I gave you of the inflammatory affections of the eye, considered apart from specific causes, disappeared as soon as we commenced the examination of the specific forms of inflammation. What, then, would it be were I to attempt to describe their various combinations;—were I, for instance, to speak of *rheumatico-scrofulous*, *catarro-scrofulous*, *catarro-rheumatic* ophthalmiæ, &c. &c.!!

In conclusion, I must again give it as my decided opinion, that all these appellations have no practical utility, and that as they only tend to render still more intricate a branch of pathology which, from the structure of the affected organ, is inevitably of a very complex nature, I think they ought to be altogether abandoned. If the profession in general were to adopt these views, and to treat inflammatory affections of the eye as simple inflammations of the tissues which enter into the formation of that organ, taking into consideration at the same time time, the constitution of the patient, ophthalmology would then be reduced to its most simple expression.

*Syphilitic Ophthalmia.*

An attentive and careful examination of the inflammatory diseases by which the eye is attacked, has shown us that those forms of ophthalmia which authors have denominated *catarrhal, arthritic, rheumatic*, and *scrofulous*, do not present any thing specific either in their symptoms or in their treatment; but this is no longer true with regard to syphilitic ophthalmia, for it is an undeniable fact that syphilis does impress on inflammatory affections of the eye peculiar characters which require a specific treatment. When, indeed, we consider, that if, in pathology, there is a specific disease, it is certainly syphilis, and that, when the economy is deeply impregnated with the syphilitic virus, all other diseases which may occur appear to be more or less modified by that virus, we cannot but acknowledge that it is perfectly rational to admit the existence of syphilitic ophthalmia.

This really specific class of inflammatory affections must not be confounded with the malady which I described under the name of "gonorrheal conjunctivitis." There is the same difference between these diseases as between a venereal chancre and a gonorrheal affection.

Some authors (M. Sichel among others) think that the iris alone is the seat of the syphilitic ophthalmia, but this is an error. The iris is oftener affected, it is true, than any other part of the eye, but the malady is also occasionally seen in other tissues. I have myself met with several cases in which the eyelids or the cornea were apparently attacked by this form of inflammation. A short time ago I had under my care a youth evidently labouring under syphilitic blepharitis. The margin of the eyelids was swollen, and presented a yellow copper-coloured appearance, the conjunctiva and the cornea being at the same perfectly healthy. Every kind of treatment had been tried during a space of six months, but without success. Suspecting the nature of the affection, I submitted him to a course of mercury, and in a short time he was completely cured.

I have also seen cases of keratitis, in which the cornea was vascuralized, and offered a peculiar copper-coloured appearance, and which had resisted every other plan of treatment, give way rapidly as soon as mercury was resorted to.

The symptoms which are given by ophthalmologists as indicating the presence of syphilitic iritis, are the following:—The iris presents a copper-coloured appearance, its tissue becomes turgid, and its anterior surface assumes a velvety aspect. At the same time the pupil, losing its regularity of shape, becomes deformed superficially and internally.

But this description is only partially correct, as the change which takes place in the form of the iris is merely a symptom of the inflammation of that membrane, and is not oftener observed internally than externally, superiorly than inferiorly. Indeed, as I have already told you, the modifications which may occur in the form of the pupil are not of the slightest avail towards distinguishing the different forms of iritis.

Soon small flakes, described by Beer under the name of *condyloma*, and by Müller under that of *crista galli*, make their appearance at the circumference of the pupil, forming, as it were, a kind of fringe. In addition to these symptoms, which may be considered characteristic of syphilitic iritis, when the inflammation is acute, there are others which generally accompany simple iritis, such as haziness of the aqueous humours, vascularization of the conjunctiva and sclerotica, and disorder of the visual functions. The pain which the patients feel is generally greater during the night than during the day.

Whenever these symptoms are present, and the patient is or has been under the influence of syphilis, we may conclude that the inflammation is of a syphilitic nature. Syphilitic iritis may be classed among secondary syphilitic affections, as it is produced by the same cause and follows the same course.

Once we have recognised the syphilitic nature of this form of ophthalmia, it becomes evident that recourse must be had to an antisyphilitic treatment. If, however, the inflammatory symptoms run high before we resort to the specific treatment of syphilis, the inflammation must be subdued by general and local bloodletting, and by the other measures which I enumerated when speaking of acute iritis. If, on the contrary, the inflammation is chronic, we may begin at once the mercurial treatment. I generally order mercurial frictions to be practised on the internal parts of the thighs, and give internally the proto-ioduret of mercury.—*London Med. Gaz.*

---

*Sir B. Brodie on the Treatment of Hysteria.*—On this head I must observe to you again on the danger of treating hysterical diseases as you would other diseases. Another object which you must have in view is to withdraw the patient's attention as much as possible from the disease. This alone will do more good than any thing else. In some cases it is better to treat them by doing nothing at all, because by employing local applications you draw the patient's attention to the seat of the affection. Where there is much pain in the part you may apply a belladonna plaster over it, or you may moisten it with equal parts of tepid rose water and camphor mixture, and you will find that this application will readily relieve the pain.—*London Lancet.*

